# Effects of the Infusion of 4% or 20% Human Serum Albumin on the Skeletal Muscle Microcirculation in Endotoxemic Rats

**DOI:** 10.1371/journal.pone.0151005

**Published:** 2016-03-04

**Authors:** Elisa Damiani, Can Ince, Fiorenza Orlando, Elisa Pierpaoli, Oscar Cirioni, Andrea Giacometti, Federico Mocchegiani, Paolo Pelaia, Mauro Provinciali, Abele Donati

**Affiliations:** 1 Anaesthesia and Intensive Care Unit, Department of Biomedical Sciences and Public Health, Università Politecnica delle Marche, Ancona, Italy; 2 Department of Translational Physiology, Academic Medical Center, University of Amsterdam, Amsterdam, The Netherlands; 3 Advanced Technology Center for Aging Research, Scientific Technological Area, IRCCS-INRCA, Ancona, Italy; 4 Institute of Infectious Disease and Public Health, Università Politecnica delle Marche, Ancona, Italy; 5 Department of Experimental and Clinical Medicine, Università Politecnica delle Marche, Ancona, Italy; University of Leicester, UNITED KINGDOM

## Abstract

**Background:**

Sepsis-induced microcirculatory alterations contribute to tissue hypoxia and organ dysfunction. In addition to its plasma volume expanding activity, human serum albumin (HSA) has anti-oxidant and anti-inflammatory properties and may have a protective role in the microcirculation during sepsis. The concentration of HSA infused may influence these effects. We compared the microcirculatory effects of the infusion of 4% and 20% HSA in an experimental model of sepsis.

**Methods:**

Adult male Wistar rats were equipped with arterial and venous catheters and received an intravenous infusion of lipopolysaccharide (LPS, serotype O127:B8, 10 mg/kg over 30 minutes) or vehicle (SHAM, n = 6). Two hours later, endotoxemic animals were randomized to receive 10 mL/kg of either 4% HSA (LPS+4%HSA, n = 6), 20% HSA (LPS+20%HSA, n = 6) or 0.9% NaCl (LPS+0.9%NaCl, n = 6). No fluids were given to an additional 6 animals (LPS). Vessel density and perfusion were assessed in the skeletal muscle microcirculation with sidestream dark field videomicroscopy at baseline (t0), 2 hours after LPS injection (t1), after HSA infusion (t2) and 1 hour later (t3). The mean arterial pressure (MAP) and heart rate were recorded. Serum endothelin-1 was measured at t2.

**Results:**

MAP was stable over time in all groups. The microcirculatory parameters were significantly altered in endotoxemic animals at t1. The infusion of both 4% and 20% HSA similarly increased the perfused vessel density and blood flow velocity and decreased the flow heterogeneity to control values. Microvascular perfusion was preserved in the LPS+20%HSA group at t3, whereas alterations reappeared in the LPS+4%HSA group.

**Conclusions:**

In a rat model of normotensive endotoxemia, the infusion of 4% or 20% HSA produced a similar acute improvement in the microvascular perfusion in otherwise unresuscitated animals.

## Introduction

Fluid resuscitation is a cornerstone in the management of septic patients; however, what the best fluid to use in this condition has not been fully established. Crystalloids are currently recommended as first fluids of choice, whereas the use of albumin is suggested for patients requiring substantial amounts of crystalloids [1]. Albumin is primarily responsible for the plasma colloid osmotic pressure and is also a natural plasma volume expander [2]. It acts as a carrier for a number of endogenous and exogenous molecules, including drugs such as antibiotics, sedatives and anticoagulants [3], and has antioxidant [4] and anti-inflammatory properties [5]. Despite these many potential advantages, clinical trials yielded conflicting results and failed to show a clear and consistent survival benefit following albumin administration in patients with severe sepsis/septic shock [6–9].

Sepsis is characterized by an enhanced activation of inflammatory and oxidative stress pathways, which leads to endothelial dysfunction and vascular hyporeactivity [10]. Impaired microvascular perfusion is increasingly recognized as a major determinant of tissue hypoxia during sepsis and is a key factor in the pathogenesis of sepsis-induced organ failure [11, 12]. For its ability to counteract oxidative and nitrosative stress, albumin may represent not only a plasma expander but also an endothelium-modulating agent. In an experimental rodent model of endotoxemia, human serum albumin (HSA) prevented endothelial dysfunction and vascular hyporeactivity [13]. Tokunaga et al. showed an increase in myocardial tissue oxygenation and ventricular contractility after the infusion of 5% rat albumin in endotoxemic rats [14]. However, the optimal concentration to use is a controversial issue. Kremer et al. [15] showed that 4% HSA administration increased survival and prevented endothelial dysfunction in endotoxemic mice, whereas HSA at higher concentrations was detrimental. These data were not confirmed by others, who showed a protective effect of hyperoncotic albumin on sepsis-induced intestinal and lung injury [16].

We hypothesized that albumin may exert concentration-dependent effects on microvascular perfusion when used for fluid resuscitation during sepsis. In the present study, we evaluated the effects of infusing either 4% or 20% HSA on the microcirculation in a rat model of endotoxemia.

## Materials and Methods

Adult (aged 10±2 months) male Wistar rats (500±50 g body weight) were used in this study. The animals were maintained on a 12-h light/dark cycle and were given free access to water and standard rat chow. All experiments were performed in accordance with ethical standards and according to national and international guidelines and were approved by the animal research ethics committee of the INRCA-IRCCS (protocol number 1CHEPT/05-13). All surgery was performed under anaesthesia and all efforts were made to minimize suffering.

### Experimental procedures

**Fig 1:**
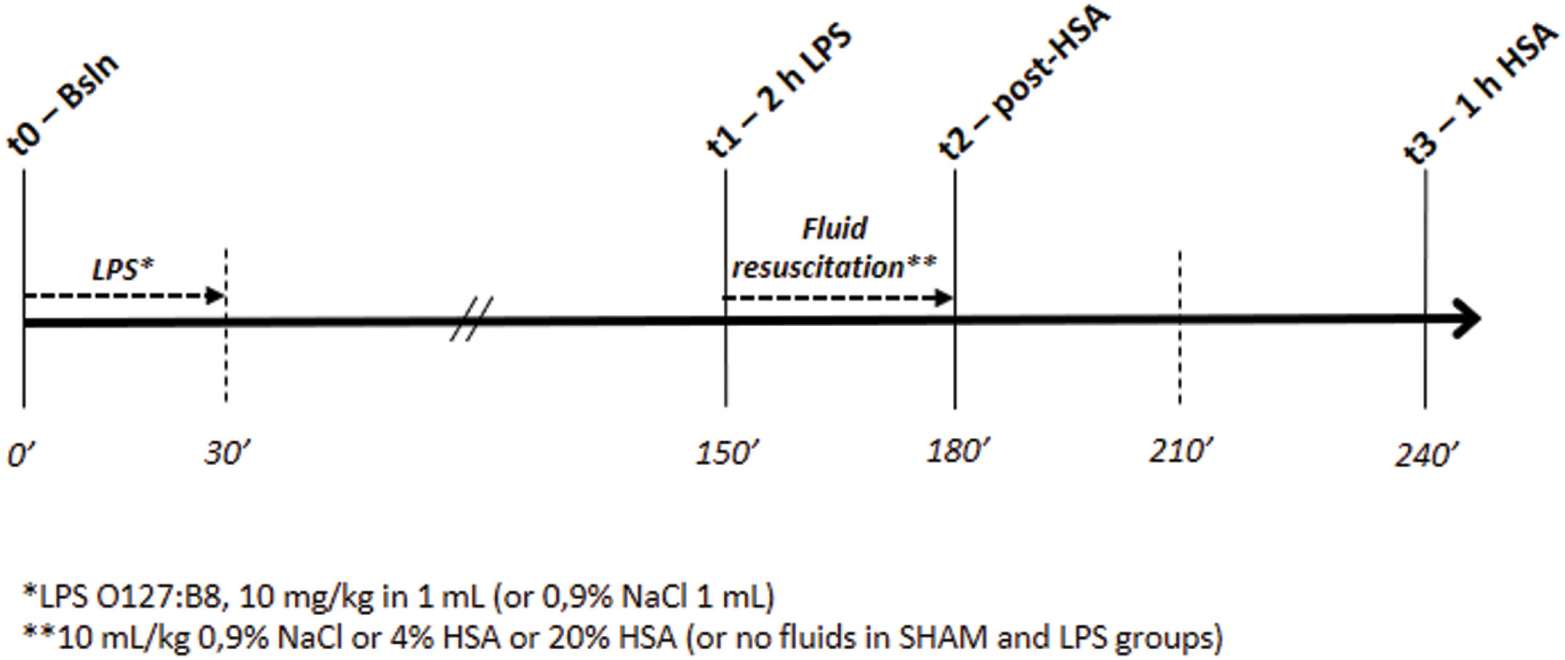
Timeline of the experimental protocol. *LPS* lipopolysaccharide, *HSA* human serum albumin.

Anesthesia was induced through intraperitoneal injection of tribromoethanol (400 mg/kg). The rectal temperature was maintained at 37°C throughout the surgical procedure by placing the rat on a heated mat. Buprenorphine (0.01 mg/kg) and meloxicam (1 mg/kg) were administered subcutaneously to all animals before surgical instrumentation and at 24 hours to ensure analgesia. The left common carotid artery and right internal jugular vein were cannulated for blood pressure monitoring/blood sampling and fluid administration, respectively. The heparinized catheters were tunnelled subcutaneously and exteriorized at the back of the neck. Six animals died during the surgical instrumentation under anesthesia (3 from severe hemorrhage during arterial cannulation, 2 from respiratory arrest, and 1 was culled for inability to obtain functional vascular accesses), resulting in a total of 36 rats used to obtain the final study sample of 30 rats. At the end of the surgical instrumentation, the animals were housed in single cages with free access to food and water until the study day. One day after surgery, anesthesia was induced with 4% isoflurane in an induction chamber. The anesthetized animal was placed on an operating table, and anesthesia was maintained with 1.2–1.5% isoflurane via a facial mask. The body temperature was monitored with a rectal probe and maintained at 37°C with a heated mat. A timeline of the experimental protocol is presented in Fig 1.

Endotoxemia was induced through intravenous infusion of lipopolysaccharide (LPS, serotype O127:B8, Sigma-Aldrich Corporation, St Louis, MO, USA) at a dose of 10 mg/kg in 1 mL over 30 minutes. An equal volume of vehicle (0.9% NaCl) was administered to a group of sham-operated animals (SHAM, n = 6). Two hours after the end of LPS infusion, rats were randomly assigned to four groups. Fluid resuscitation was performed over 30 minutes with an intravenous infusion of either 10 mL/kg 4% HSA (Albital^®^, Kedrion S.p.A., diluted in 0.9% NaCl) (LPS+4%HSA, n = 6), 10 mL/kg 20% HSA (LPS+20%HSA, n = 6) or 10 mL/kg of control fluid (0.9% NaCl, LPS+0.9%NaCl, n = 6). Fluid infusion was then discontinued. No fluids were given to an additional six animals (LPS). At the end of the experiments or upon the occurrence of pre-specified humane endpoints, such as persistent labored breathing, extreme bradycardia and/or persistent extreme hypotension indicating impending death, rats were sacrificed with an overdose of pentobarbital.

### Measurements

In all animals, measurements were taken at baseline (t0 –Bsln), 2 hours after the end of LPS infusion (t1–2 h LPS), at the end of fluid resuscitation (t2 –post-HSA) and 1 hour after the end of fluid resuscitation (t3) (Fig 1). The arterial line was connected to a pressure transducer to monitor the mean arterial pressure (MAP) and heart rate (HR) (DASH 5000, GE Healthcare). A sidestream dark field (SDF) videomicroscopy system (Microscan, Microvision Medical, Amsterdam, NL) was used to evaluate the skeletal muscle microcirculation. The Microscan is a hand-held video microscope system enabling the real-time in vivo evaluation of blood flow in microvascular beds. Details on this technique have been described elsewhere [17]. Under isoflurane anesthesia, a 2-cm skin incision was made on the medial side of the left hind limb. The connective tissue was carefully removed, and the perimysium was separated from the muscle by blunt dissection to minimize tissue damage and bleeding. The lens of the microscope was covered with a sterile disposable cap and applied to the muscle surface. A supportive device was used to enhance stability during image acquisition and to help avoid pressure artifacts [18]. Five videos from different areas (at least 10 seconds per site) were recorded at each time point with adequate focus and contrast. Poor quality images were discarded, and 3 videos per time-point were analyzed using a dedicated software (Automated Vascular Analysis 3.0, Microvision Medical, Amsterdam, NL). The total vessel density (TVD), perfused vessel density (PVD), De Backer score, proportion of perfused vessels (PPV), microcirculatory flow index (MFI), flow heterogeneity index (FHI) and blood flow velocity (BFV) were calculated as previously described [19–21]. Arterial blood samples (0.5 mL) were obtained at t2, and the serum was stored at -70°C for the subsequent measurement of endothelin-1 (Elabscience Biotechnology, Wuhan, China). The serum was treated according to the assay manufacturer’s instructions and diluted appropriately. All samples and standards were processed in duplicate.

### Statistical analysis

Statistical analysis was performed using GraphPad Prism Version 5 (GraphPad Software, La Jolla, CA, USA). The study was powered to detect a 20% difference in MFI between the groups at t2 (post fluid resuscitation) with a two-tailed alpha level of 0.05 and a beta level of 0.2 (80% power) and expecting an MFI of 2.8 ± 0.2 in sham-operated rats [22]. Data are expressed as the median [1^st^-3^rd^ quartile]. The Kruskal-Wallis test with Dunn’s post hoc test for multiple comparisons was used to analyse the data. The Friedman test with Dunn’s post hoc test was used for the comparison between time points in the same group. The alpha level of significance was set a priori at 0.05.

## Results

The mortality rate for each experimental group was SHAM 0/6, LPS 4/6, LPS+0.9%NaCl 4/6, LPS+4%HSA 2/6, LPS+20%HSA 3/6. Three animals in total were euthanized before reaching the experimental endpoint for the occurrence of persistent labored breathing and gasping (humane endpoint), with one in the LPS group, one in the LPS+0.9%NaCl and one in the LPS+20%HSA. All the other animals died spontaneously from cardiovascular collapse with a rapid drop of mean arterial pressure and cardiac arrest. One rat in the LPS group died after t1. All animals in the other groups survived until the end of fluid resuscitation. At t3 (1 h post-HSA), 4 and 3 rats were alive in the LPS+4%HSA and LPS+20%HSA groups, respectively. Only 2 animals were alive in the LPS and LPS+0.9%NaCl groups (these were excluded from the between-group comparison at t3).

No significant variations in MAP were observed over time in any of the groups (Table 1). HR was elevated in the LPS+4%HSA group at t2 (p<0.01 versus t0-baseline, p<0.05 versus LPS, p<0.01 versus LPS+0.9%NaCl).

**Table 1:** Variations in mean arterial pressure and heart rate throughout the experiment in all groups.

	*Mean Arterial Pressure (mmHg)*			
*Groups*	*t0—Baseline*	*t1–2 h post-LPS*	*t2 –post-HSA*	*t3–1 h post-HSA*
SHAM	126 [95–142]	118 [84–137]	123 [88–136]	134 [101–149]
LPS	131 [107–136]	113 [76–138]	121 [53–148]	-
LPS+0.9%NaCl	121 [95–134]	114 [61–140]	100 [71–131]	-
LPS+4%HSA	124 [110–138]	110 [82–140]	89 [82–129]	77 [40–135]
LPS+20%HSA	118 [101–127]	110 [83–114]	88 [78–118]	71 [58–85]
	*Heart Rate (bpm)*			
*Groups*	*t0—Baseline*	*t1–2 h post-LPS*	*t2 –post-HSA*	*t3–1 h post-HSA*
SHAM	293 [236–309]	280 [259–335]	287 [249–320]	276 [259–332]
LPS	296 [240–354]	295 [251–299]	286 [249–305]	-
LPS+0.9%NaCl	346 [316–371]	300 [260–334]	350 [293–396]	-
LPS+4%HSA	306 [285–365]	340 [308–365]	397 [355–407]*^##^^aa^	365 [305–404]
LPS+20%HSA	281 [217–342]	320 [284–343]	322 [274–354]	257 [228–315]

Six animals per group were studied. One rat in the LPS group died after t1, leaving 5 animals in this group at t2 (post HSA). Data are expressed as the median [interquartile range]. *LPS* lipopolysaccharide; *HSA* Human Serum Albumin.

*p<0.05, versus LPS;

^##^ p<0.01, versus LPS+0.9%NaCl;

^aa^ p<0.01, versus t0-Baseline.

**Fig 2:**
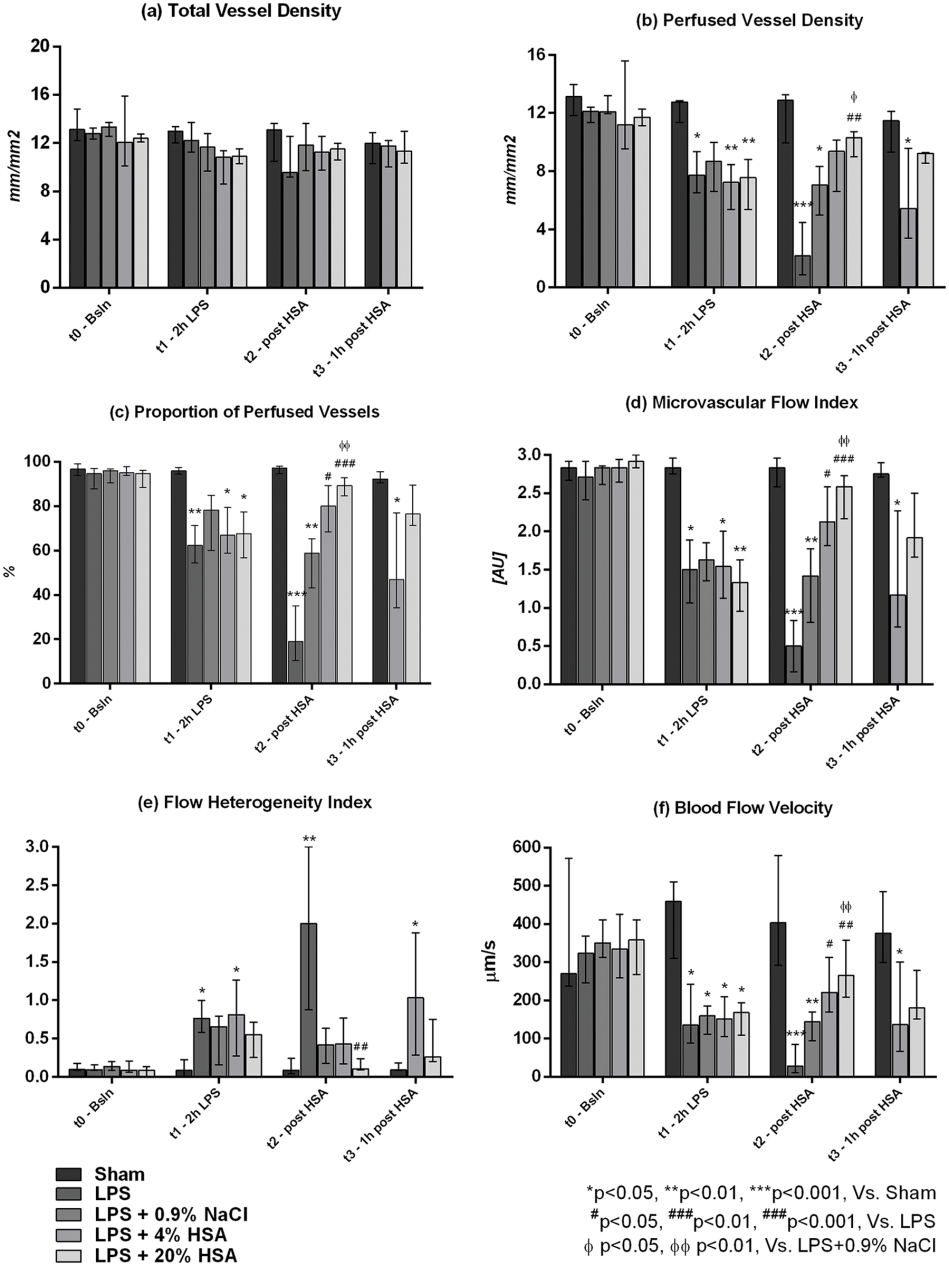
Variations in microcirculatory parameters throughout the experiment: (a) total vessel density, (b) perfused vessel density, (c) percentage of perfused vessels, (d) microcirculatory flow index, (e) flow heterogeneity index, (f) blood flow velocity. Six animals per group were studied. One rat in the LPS group died after t1, leaving 5 animals in this group at t2 (post-HSA). Two rats died after t2 in the LPS+4%HSA and three rats died after t2 in the LPS+20%HSA groups, leaving 4 and 3 rats at t3 respectively. Since only 2 animals were alive in the LPS and LPS+0.9%NaCl groups at t3, these were excluded from statistical analysis at this time point and are not presented in the graph. Data are expressed as median [interquartile range]. *LPS* lipopolysaccharide; *HSA* Human Serum Albumin. *p<0.05, **p<0.01, ***p<0.001, versus SHAM; ^#^p<0.05, ^##^p<0.01, ^###^p<0.001, versus LPS; ^ɸ^ p<0.05, ^ɸɸ^ p<0.01, versus LPS+0.9%NaCl.

Microcirculatory assessment revealed a significant impairment in vessel perfusion and flow quality in endotoxemic animals at t1 (2 h LPS), despite a relatively preserved vessel density (Fig 2).

The TVD was similar in all groups at all time-points. The PVD, PPV, MFI and BFV were decreased at t1 in the LPS, LPS+0.9%NaCl, LPS+4%HSA and LPS+20%HSA groups compared to the sham-operated rats. The FHI was increased (p<0.05 for LPS and LPS+4%HSA versus SHAM).

**Fig 3:**
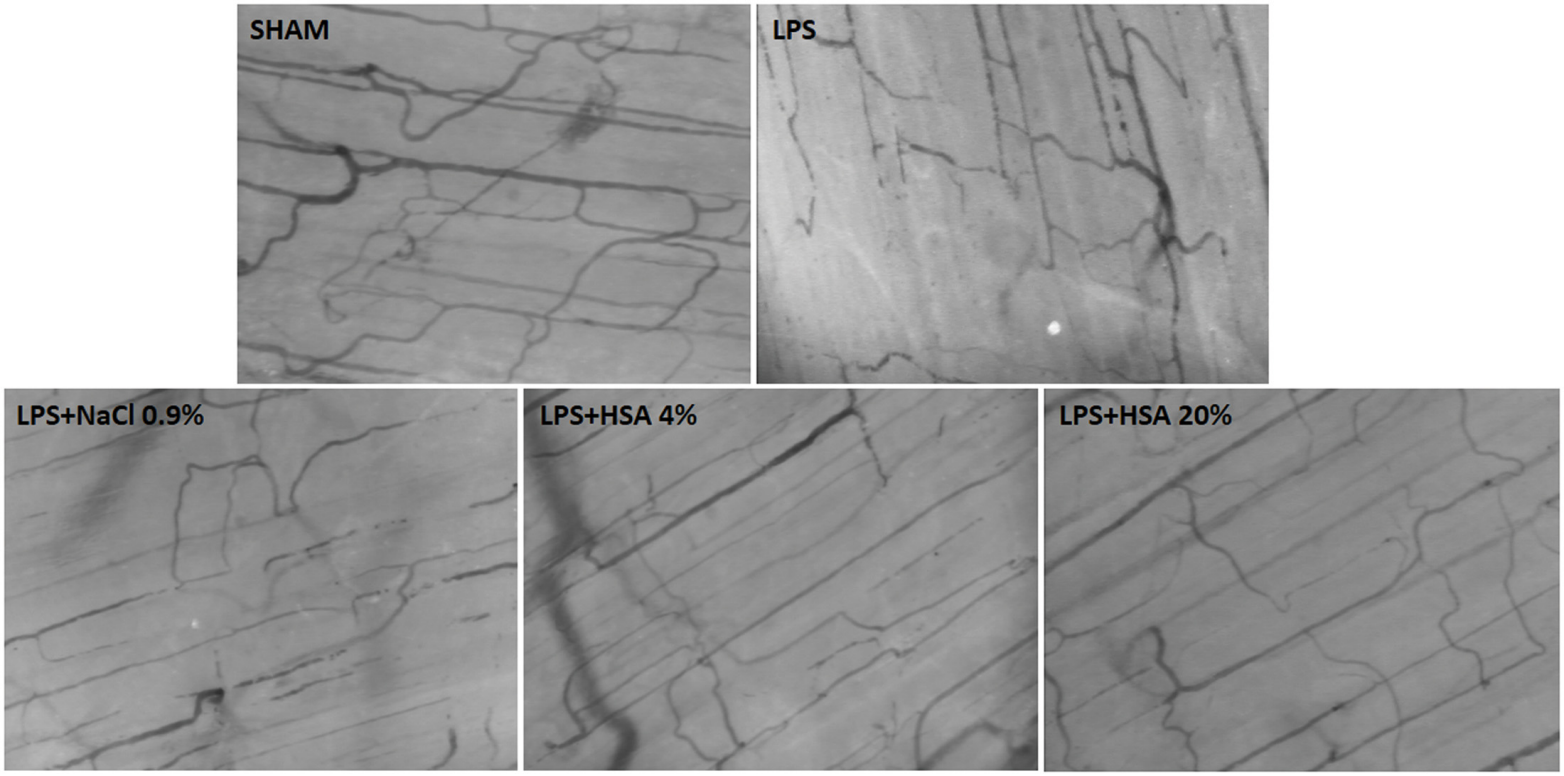
Examples of the skeletal muscle microcirculation at t2 (end of fluid resuscitation) for all the study groups.

At the end of fluid resuscitation (t2, Figs 2 and 3), microvascular perfusion was improved in animals resuscitated with HSA but remained altered in those receiving 0.9% NaCl. The PVD, PPV, MFI and BFV were significantly elevated at t2 in LPS+4%HSA (p<0.05 versus LPS) and LPS+20%HSA groups (p<0.01 versus LPS), whereas these parameters remained significantly lower in the LPS+0.9%NaCl group compared to the sham-operated controls (p<0.05 for PVD, p<0.01 for PPV, MFI and BFV). Animals in the LPS+20%HSA group showed better microvascular perfusion compared to those in the LPS+0.9%NaCl group (p<0.05 for PVD, p<0.01 for PPV, MFI and BFV), but no significant differences were found between the LPS+4%HSA and LPS+20%HSA groups.

One hour after the end of fluid infusion (t3, Fig 2), the PVD, PPV, MFI and BFV were significantly reduced in the LPS+4%HSA group compared to the sham-operated animals (p<0.05), and the FHI was elevated (p<0.05). By contrast, animals in the LPS+20%HSA group continued to show similar microcirculatory parameters as the SHAM.

The serum levels of endothelin-1 measured at t2 did not significantly differ between the groups (SHAM: 18.3 [4.6–27.2] pg/mL, LPS: 11.6 [8.2–28.2] pg/mL, LPS+0.9%NaCl: 23.6 [4.2–34.6] pg/mL, LPS+4%HSA: 19.5 [14.8–28.1] pg/mL, LPS+20%HSA: 24.3 [18.5–30.5] pg/mL, p = 0.765).

## Discussion

We constructed a rat model of endotoxemia in which severe microcirculatory alterations occurred despite the absence of significant variations in MAP. In our study, the infusion of either 4% or 20% HSA in otherwise unresuscitated endotoxemic rats restored skeletal muscle microvascular perfusion. Although the preserved microcirculatory flow 1 hour after the end of infusion suggests a more stable effect of 20% HSA as compared to 4% HSA, a clear superiority could not be established due to the small number of animals at this time point.

Sepsis is characterized by an impaired capacity to increase tissue oxygen extraction, primarily depending on the maldistribution of capillary blood flow and microcirculatory dysfunction [12, 23]. Clinical studies showed that the persistence of sepsis-induced microvascular alterations is associated with a worse outcome, whereas an improvement in small-vessel perfusion was only observed in eventual survivors [11, 24]. Microcirculatory disturbances have been extensively described in experimental models of sepsis and endotoxemia [25] and appeared to be early and relatively independent of macro-hemodynamic changes [22, 26]. In our study, endotoxemia impaired microvascular blood flow, leading to a reduction in blood flow velocity and perfused vessel density, and caused a maldistribution of capillary flow, as indicated by the elevation in FHI. Fluid resuscitation with HSA restored the microcirculatory flow to normal values as observed in non-endotoxemic animals by increasing the blood flow velocity and the number of perfused vessels. These data are consistent with those of previous studies. In a mouse model of endotoxic shock, HSA modulated nitric oxide production and the activation of inflammatory pathways by decreasing the up-regulation of inducible nitric oxide synthase and nuclear factor-kB in the aorta, and this was associated with improved endothelial dysfunction and reduced vascular hyporeactivity [13]. Other mechanisms for the protective effect of HSA on the microcirculation include anti-oxidant properties, such as directly scavenging free radicals [27] or interacting with neutrophils [28], with a potential beneficial effect on the endothelial glycocalyx [29].

In a mouse model of endotoxemia, only 4% HSA increased survival and modulated the LPS-induced increase in glutathione (GSH) and endothelin-1, showing anti-oxidant and vascular protective effects, whereas 20% HSA was potentially pro-oxidant [15]. By contrast, in a rat model of sepsis using cecal ligation and puncture, Chian et al. showed that 25% HSA but not 5% HSA counteracted sepsis-induced hypotension and vascular hyporeactivity, reduced pulmonary oxidative and nitrosative stresses and improved histopathologic changes in the lungs [16]. In our model, 4% and 20% HSA produced a similar improvement in microvascular perfusion, with no clear superiority of one dose over another at the end of the infusion. In contrast to Kremer et al. [15], we did not find any difference in serum endothelin-1 between the groups at the end of the fluid infusion. Of note, however, only animals resuscitated with 20% HSA had significantly better microcirculatory parameters at the end of the infusion compared to those receiving 0.9% NaCl as a control fluid. The infusion of 20% HSA may have produced a more stable improvement in microvascular perfusion, because this remained similar to that of sham-operated rats 1 hour after the end of fluid infusion, whereas alterations in microvascular flow and density reappeared in animals resuscitated with 4% HSA. Nonetheless, these data must be interpreted with caution, since the small number of animals in these two groups at this time point prevents obtaining definite conclusions.

The plasma volume expanding effect of HSA may play a major role in our model in which no other fluids were administered. Unrecognized hypovolemia may be at least partly responsible for the microvascular alterations observed, and the beneficial effect of 20% HSA may reflect a greater impact of the infusion of hyperoncotic fluids on the microvascular convective flow under a condition of volume depletion [30]. However, the absence of significant differences in MAP between the groups rules out a possible role of variations in perfusion pressure in driving the microvascular changes observed after fluid resuscitation. The infusion of 0.9% NaCl was not intended to induce a similar plasma expanding effect as HSA, because the same infusion volume was used. The 0.9% NaCl was not tested as a possible alternative treatment but was administered to have a group of sham-resuscitated endotoxemic rats because we focused on the comparison between two different concentrations of HSA.

Our model was characterized by high severity and mortality, with only 2 rats of 6 surviving for more than 2 hours in the LPS group. LPS administration did not produce a progressive decrease in MAP over time but rather a rapid drop shortly before death. This was a short-term model to evaluate the effects of different fluid resuscitation regimens on LPS-induced microcirculatory disturbances. It is possible that we were unable to detect changes in endothelin-1 levels due to the short time frame of our experiment. Longer-term and more realistic models of sepsis, such as cecal ligation and puncture, could be more suitable for investigating the pleiotropic effects of albumin, particularly its anti-oxidant and anti-inflammatory properties.

Clinical trials, despite supporting the safety of HSA administration, failed to provide sufficient evidence in favor of the use of albumin as the resuscitation fluid of choice for patients with severe sepsis and septic shock [6–9]. In the recently published Albumin Italian Outcome Sepsis (ALBIOS) study, the administration of 20% HSA as a replacement therapy did not reduce either 28- or 90-day mortality in a population of patients with severe sepsis; however, a subgroup analysis revealed a significant survival benefit among patients with septic shock [31]. Another multicenter randomized controlled trial showed that the early administration of 20% HSA versus 0.9% NaCl did not yield any survival benefit in patients with septic shock, although it was associated with a reduction in catecholamine requirements [32]. The potential beneficial effects of albumin administration may not be manifested in all patients with sepsis, but are likely to be more pronounced in certain subgroups (higher severity, greater inflammatory response). Clinical studies evaluating the effects of HSA infusion on the microcirculation during severe sepsis/septic shock are lacking and may contribute to the identification of subgroups of patients who may benefit the most from albumin administration, in addition to clarifying the micro-hemodynamic effects of different dose regimens.

Our study has several limitations. First, we applied a model of endotoxemia that cannot be considered an accurate model of human sepsis but rather a well-characterized model of the acute systemic inflammatory response occurring during sepsis. Therefore, although we propose that LPS infusion reproduced similar microcirculatory alterations as those that occur during real sepsis, our results cannot be directly extrapolated to the clinical setting. By contrast, an advantage of our model was the use of adult animals aged 10±2 months (an age that more closely reproduces the mean age of patients with sepsis), whereas the vast majority of experimental studies on small rodents are performed on animals aged 6–16 weeks, corresponding to a human age of 10–17 years [30]. Second, we could not demonstrate that the observed improvement in microvascular perfusion with HSA was associated with increased tissue oxygenation. Whether fluid resuscitation improves perfusion by increasing cardiac output, the administration of solutions with no O_2_-carrying capacity may lead to hemodilution and result in no net increase in tissue O_2_ delivery. In rat models of endotoxemia or hemorrhagic shock, fluid resuscitation was unable to improve renal oxygenation [33], which could be partially restored only with blood transfusions [34]. Unfortunately in our study, measures of regional (tissue PO_2_ levels) or systemic (O_2_ delivery, lactate levels) oxygenation were not collected. A third limitation is that we did not evaluate the effects of HSA infusion on oxidative/nitrosative stress or inflammatory pathways; therefore, we cannot determine whether the observed improvement in the microcirculation was only dependent on a plasma expanding effect or whether other mechanisms were also involved. In addition, our model was not designed to evaluate survival and we could not determine whether the restoration of microvascular perfusion was associated with a reduction in mortality. Finally, we assessed a peripheral microvascular bed (skeletal muscle) and cannot exclude the possibility that the microcirculatory response to HSA was different in other organs. We selected the skeletal muscle of the hind limb for ease of access, stability during image acquisition and a microvascular architecture that lends itself to the assessment of flow and vessel density. Moreover, similar alterations in vascular responsiveness were observed in the mesenteric and skeletal muscle microcirculation in a rat model of sepsis [35].

## Conclusions

In a rat model of normotensive endotoxemia, the infusion of 4% or 20% HSA restored microvascular perfusion in otherwise unresuscitated animals. A more stable microvascular improvement with 20% HSA cannot be excluded, although our study was underpowered to demonstrate a superior effect of 20% over 4% HSA 1 hour after the end of fluid infusion.
